# The tumour histopathology “glossary” for AI developers

**DOI:** 10.1371/journal.pcbi.1012708

**Published:** 2025-01-23

**Authors:** Soham Mandal, Ann-Marie Baker, Trevor A. Graham, Konstantin Bräutigam

**Affiliations:** 1 Centre for Evolution and Cancer, Institute of Cancer Research, London, United Kingdom; 2 Data Science Team, Institute of Cancer Research, London, United Kingdom; Origin Bioinformatics, CANADA

## Abstract

The applications of artificial intelligence (AI) and deep learning (DL) are leading to significant advances in cancer research, particularly in analysing histopathology images for prognostic and treatment-predictive insights. However, effective translation of these computational methods requires computational researchers to have at least a basic understanding of histopathology. In this work, we aim to bridge that gap by introducing essential histopathology concepts to support AI developers in their research. We cover the defining features of key cell types, including epithelial, stromal, and immune cells. The concepts of malignancy, precursor lesions, and the tumour microenvironment (TME) are discussed and illustrated. To enhance understanding, we also introduce foundational histopathology techniques, such as conventional staining with hematoxylin and eosin (HE), antibody staining by immunohistochemistry, and including the new multiplexed antibody staining methods. By providing this essential knowledge to the computational community, we aim to accelerate the development of AI algorithms for cancer research.

## Introduction

Histopathology—the microscopic analysis of tissue samples to diagnose and study diseases—is the mainstay of cancer diagnosis, and most clinical practices revolve around expert human pathologists examining very thin tissue slices (“sections”) mounted on glass slides under traditional light microscopes.

The last decades have experienced a huge rise in the application of bioinformatics, artificial intelligence (AI), and machine learning (ML) in cancer research [1–3]. A plethora of AI tools have been developed specifically for histopathology, which aim to improve its diagnostic reliability and accuracy [4–6]. Tissue, i.e., the sum and interplay of cells, and cellular morphologies are the key variables in histopathology [7] and are the foundation for any supervised modelling attempt. Thus, we believe that successful AI development requires an understanding of tumour histology.

Building accurate AI models for medicine is an interdisciplinary task [8] and requires a complement of different expertise, i.e., computational and mathematical skills combined with clinical knowledge. This is often hampered by computer scientists and biologists/expert healthcare professionals not physically working in the same environment, where they cannot easily access the other’s expertise. The lack of interdisciplinary communication results in significant inefficiencies and misunderstandings, leading many AI models to remain prototypes rather than being integrated into clinical practice [9,10]. In light of the rise of AI in the clinic, teaching medics AI basics has become essential [11]; here, we propose that a similarly reciprocal understanding of biology is important for AI developers to build better models.

Recent advancements in AI research have significantly automated a range of diagnostic (defining disease), predictive (predicting the response to a certain treatment [12]), and prognostic (stratifying patients at risk and determining outcome/patient prognosis) tasks in oncology [13–15]. However, these models often operate as “black boxes,” providing little transparency regarding their decision-making processes. Physicians, on the other hand, base their diagnoses on well-defined biological features and established criteria [16], especially in the case of histopathologists, whose cornerstone is tissue and cell morphology [17]. Therefore, the interpretability of both the design and the results of AI-based methods are crucial for gaining the trust and acceptance of the medical community and accelerating the clinical implementation of in-silico approaches. A good level of understanding of tumour biology instructs AI developers to incorporate relevant biological features into their computational models [18]. This understanding not only improves the accuracy and relevance of the models but also facilitates the presentation of results in a manner that physicians can validate and understand. For example, a study on early-stage oestrogen receptor-positive (ER+) breast cancer demonstrated how considering relevant biological features is crucial in the development of AI methods for survival prediction [19]. The method described in this work leveraged understanding of nuclear pleomorphism (variance in the appearance of the cell nucleus), which is a crucial factor in breast cancer grading.

This article aims to bridge the gap between the AI development and the translation to routine clinical application by emphasising the importance of relevant biological knowledge, which would be helpful in enhancing model interpretability and the subsequent clinical validation. Only solid biological understanding would enable modellers to define sets of relevant features and implement their morphological properties into algorithms.

While recently published literature aims at bestowing (cancer) healthcare professionals with expertise on AI, e.g., developing image analysis and modelling skills for clinicians [20,21], the opposite—giving AI developers an understanding of cancer histopathology fundamentals—is rare. Thus, in this work we introduce some of the essential concepts of tumour histopathology—the most frequent cell types, the concepts of “neoplasia,” “tumours,” and the tumour microenvironment (TME). We also illustrate routine histopathology protocols and special stainings that provide the visual representation of the above concepts.

## Concepts of tumour histopathology and representative examples of modelling them

In order to model disease, firstly, a solid understanding of cell types, their physiological function, overall architecture and interplay with other cells is necessary. Parameters for image analysis and neural network training are best derived by applying knowledge of their defining morphology and distinct, if not unique, individual features (the common proverb of “the eyes can’t see what the mind doesn’t know” applies). In the first section, we introduce different cell types and the concept of “neoplasia.”

### Morphological diversities in cell types and tissues

**Fig 1:**
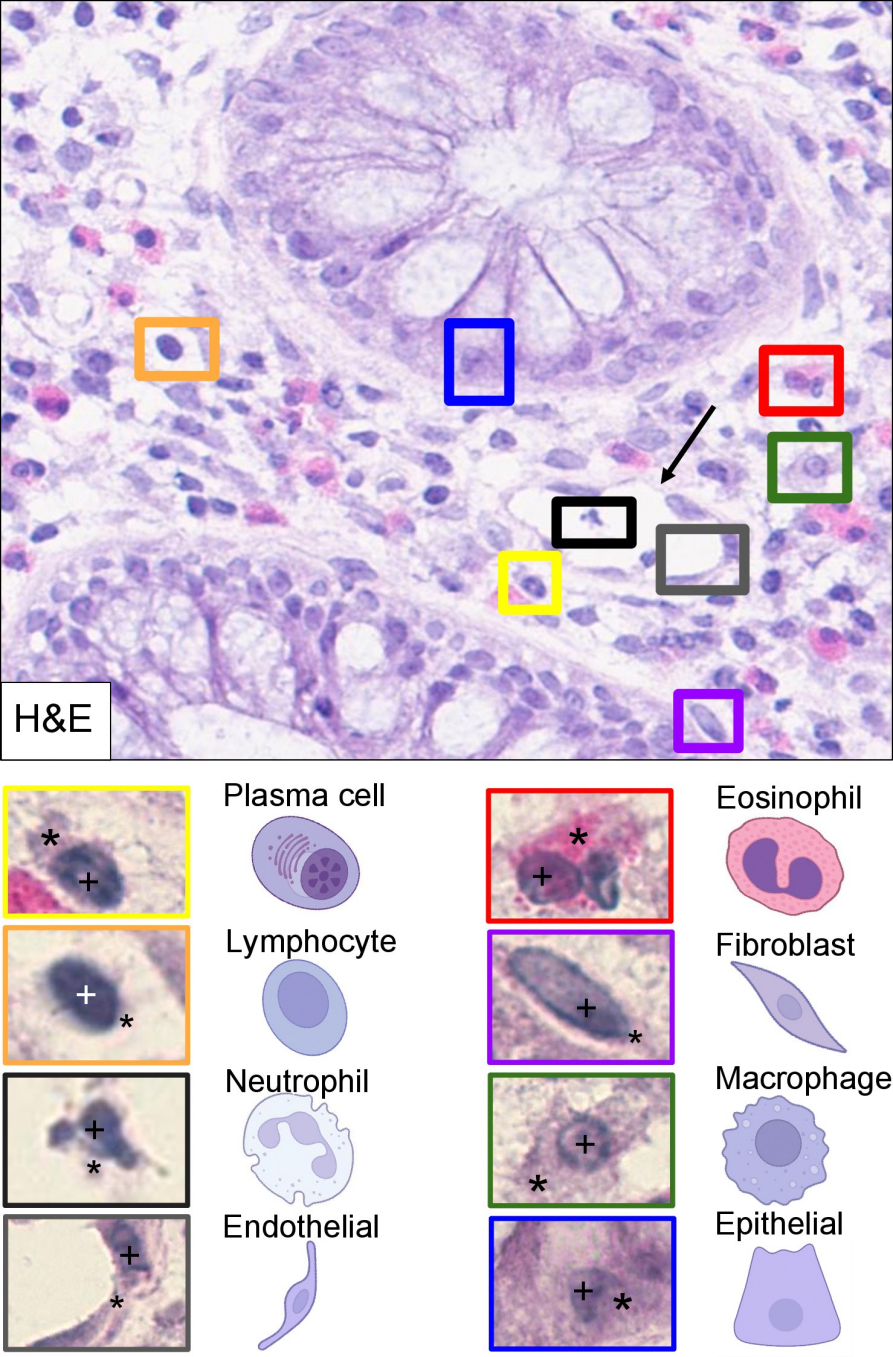
The morphological spectrum of different cell types (representative example using HE staining of a human colon). Regions of interest with different cell types are highlighted with coloured boxes, i.e., inflammatory cells (lymphocytes, macrophages, eosinophils, neutrophils, and plasma cells), epithelial cells (here, an enterocyte forming the cellular unit of crypts), endothelium, and stromal fibroblasts. Prominent cytoplasm (asterisk) in a representative macrophage. See **Table 1** and section “The hematoxylin and eosin (HE) stain” for further description. Overview scanned on a *Hamamatsu Nanozoomer* whole slide scanner (Hamamatsu Photonics, Hamamatsu, Japan), magnification: 40×; individual cell types captured on a *Zeiss Axio Imager Z2* microscope platform (Carl Zeiss AG, Oberkochen, Germany), magnification: 63×. *Arrow*: Blood vessel lined with endothelial cells (grey box). *Asterisks*: Cytoplasm. *Crosses*: nucleus. Partly created with *BioRender*.*com*.

Most human cells consist of nucleus and cytoplasm (**Fig 1**), both of which are organised into different compartments and organelles and surrounded by the cell membrane. The size of the nucleus exhibits a considerate amount of variability. Normal cells mostly display smooth nuclear contours and smaller size. Cancerous cells, on the other hand, tend to exhibit larger and pleomorphic (i.e., bizarre looking) nuclei with a prominent nucleolus (the spherical site for ribosome biogenesis). Further, there is variation in nuclear shape among different cell types. Fibroblasts, which are key components of connective tissue, have a spindle shape, whereas epithelial cells tend to be more round or oval (**Fig 1**). Second, the cytoplasm varies significantly in size and composition. Eosinophilic granulocytes typically feature a bilobed (two-lobed, spectacle-shaped) nucleus, while macrophages can be recognised by their large cytoplasm (**Table 1**). Overall, standard cellular morphology reveals distinct sets of features to build AI models upon for cell phenotyping. Usually, these AI models [22,23] consist of 2 sub-models, one for cell segmentation and the other for cell classification using the segmentation results. Recently, substantial progress has been made to develop unified models [24,25] for cell segmentation and classification simultaneously. However, cell phenotyping still faces significant challenges, which include but are not limited to the scarcity of annotated large scale data sets, the significant morphological heterogeneity within cell types, and the complex spatial relationships between cell types and their microenvironment [26].

**Table 1:** Cell types, their morphology (Fig 1), detection, and disease association. *CD*, Cluster of Differentiation; *HE*, hematoxylin and eosin; *MUM1*, Multiple Myeloma Oncogene 1; *SMA*, smooth muscle antigen; *TME*, tumour microenvironment.

Cell type	Distinguishing morphological, cytological, and spatial characteristics	Immunohistochemistry	Disease setting
Epithelial cell	Distinct shape (polyhedral geometry), pink cytoplasm, ovoid shape	Cytokeratin/s	e.g., colorectal adenoma, carcinoma (**Fig 2**)
Lymphocyte	Small, round, very intense stained large nuclei (condensed chromatin), scant amount of cytoplasm. Usually scattered but can aggregate (e.g., in “follicles”). Can cluster at inflammation sites	e.g., CD3, CD4, CD8, CD20, CD45, FOXP3 (**Table 3**)	e.g., lymphoma, (chronic) inflammation, tumour-infiltrating lymphocytes (TILs)
Neutrophil	Multi-lobed nucleus and (pale looking) granular cytoplasm. Scattered, can cluster at inflammation sites	e.g., Myeloperoxidase (MPO)	e.g., acute inflammation, tumour-associated neutrophils (TANs)
Eosinophil	Bi-lobed nucleus, large and bright red-stained granules. Scattered, can cluster at inflammation sites	e.g., Major basic protein	e.g., acute and chronic inflammation, Eosinophilic Pneumonia, TME component [78]
Fibroblast	Spindle-shaped, elongated nucleus. Scattered, part of connective tissue	e.g., Vimentin	e.g., fibrosis, cancer-associated fibroblasts (CAFs)
Endothelial cell	Elongated, and flattened, clear cytoplasm, lining the lumen of (blood and lymphatic) vessels	e.g., CD31, CD34, D2-40, ERG	e.g., part of the TME (microvessels), neoplastic vasculature
Macrophage	Abundant cytoplasm, large size, phagocytosed particles	e.g., CD11b, CD68, CD163	Tumour-associated macrophages (TAMs), Whipple disease, Rosai–Dorfman disease, etc.
Plasma cell	Eccentric nucleus, “clock-face” chromatin, abundant cytoplasm	e.g., CD38, CD138, MUM1	e.g., multiple myeloma, chronic inflammation
Mast cell	Relatively large ovoid cells, cytoplasmic granules, central nucleus	e.g., cKIT (CD117), Tryptase	e.g., mastocytosis

Human tissues, i.e., functional units of synergistically working cells, are composed of collections of cells that in a non-diseased state have an ordered arrangement in space. Roughly, tissue can be subsumed into 2 major compartments; parenchyma, i.e., the functional part composed of specialised cells, and stroma, i.e., the supporting part, mainly connective tissue, extracellular matrix and (micro)vessels. While stroma is morphologically similar across tissue types, the architecture of the parenchyma can have drastic differences. As an example, breast parenchyma consists of lobules and ducts for lactation, whereas parenchyma in the heart is mainly cardiac muscle. In short, tissue function defines the composition of the parenchyma and vice versa.

### What are “tumours”?

“Tumour” (Latin for “swelling”) is an ill-defined term, in principle designating an increase in tissue volume. It refers to a neoplastic process (“neoplasia” being the abnormal and excessive growth of cells and tissue), whose biological “potential” is in most cases dichotomously classified as either benign (localised without metastatic potential, e.g., a minute hyperplastic polyp in the colon) or malignant (invading neighbouring tissue and/or moving to distant organs, e.g., colorectal cancer). The cells of origin for a neoplasia can be classed as epithelial, lymphoid (blood cells), or mesenchymal (connective tissue). Understandably, a large number of AI models focus on the most frequent cancer types [27], which are epithelial in origin and solid, i.e., mass-forming. Non-solid neoplasia are, for instance, cancers of the blood system, which are not localised and not confined to a single organ, e.g., leukemia where neoplastic cells are in circulation in the blood. In the following, we concentrate on solid neoplasia, due to its epidemiologic relevance and localised anatomy.

**Fig 2:**
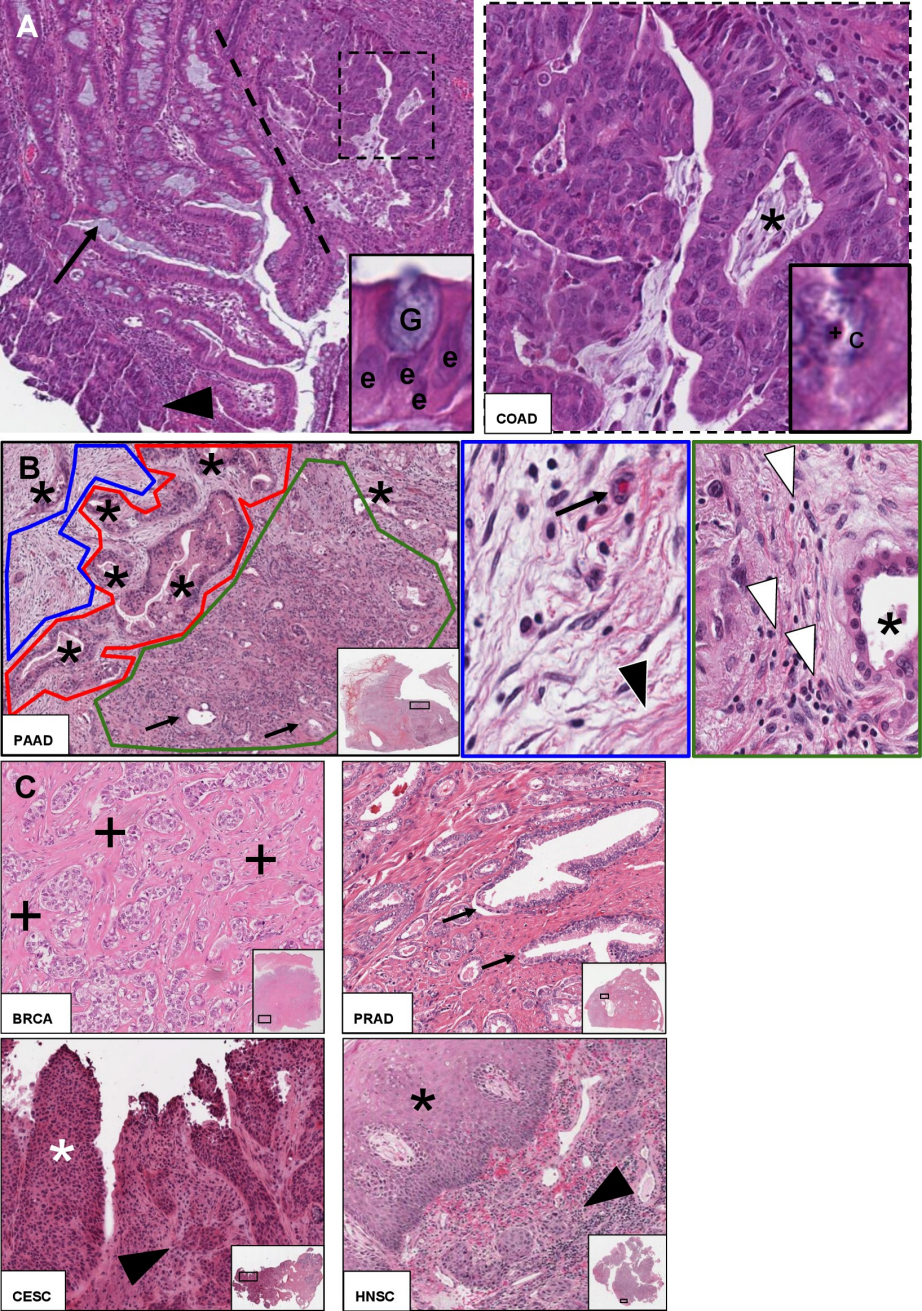
The morphology of “cancer,” HE. (**A**) Nonneoplastic epithelium (left) versus adenocarcinoma glands/cells (right, separated by dotted line) in a *The Cancer Genome Atlas* (TCGA) sample of colorectal adenocarcinoma (COAD, *TCGA-A6-2678*). Dashed *inset* (corresponding to the dashed rectangle): Higher magnification showing malignant COAD glands with malignant nuclear features and a focus of “dirty” necrosis within the cancer gland (asterisk). *Insets*: On the one hand, enterocytes (e, colonic epithelium) with regular contours, polarised towards the lumen with a goblet (mucus-containing) cell (G) on top. On the other hand, cancer cell (c) with a prominent nucleolus (cross), large size and irregular contours. *Arrow*: Regular colonic crypts, rich in goblet cells (arrow on goblet cell). *Arrowhead*: High-grade dysplasia at the surface mucosa. (**B**) Tumour parenchyma (red) and tumour stroma (blue, green) in pancreatic adenocarcinoma (PAAD, *TCGA-2J-AAB6*). PAAD glands are surrounded by dense “desmoplasia” (blue, inset) with pinkish collagen deposition (black arrowhead) and immune cell infiltrate (green, inset), mainly lymphocytes (white arrowheads) among microvessels (arrows). (**C**) Phenotype of invasion. From left to right: Invasive breast cancer (BRCA) clusters (BRCA, *TCGA-3C-AALJ*), prostatic adenocarcinoma (PRAD, *TCGA-EJ-7792*) glands with loss of basal cell layer (*arrows*: benign prostatic parenchyma), invasive squamous cell carcinoma of the cervix (CESC, *TCGA-C5-A7XC*) with adjacent high-grade squamous intraepithelial lesion (HSIL, white asterisk) and of the head and neck region (HNSC, *TCGA-BA-4076*). See **Table 2** and section “The hematoxylin and eosin (HE) stain” for further description. *Asterisks*: Surface squamous epithelium, *arrowheads*: invasive SC. *Crosses*: Extracellular matrix/stroma between BRCA clusters.

Histologically, solid neoplasia is composed of the tumour parenchyma, i.e., the neoplastic cells themselves, and the tumour stroma (**Fig 2A** and **2B**). The tumour stroma has gained more and more attention in cancer research [28], and is mainly composed of cells of the tumour microenvironment (TME, see below), extracellular matrix (structural and specialised proteins surrounding units of cancer cells), and connective tissue (mainly collagen fibres, fibroblasts, and microvessels).

### What is “tumour invasion” and its predecessors?

Tumour invasion is the critical step to a malignant phenotype in epithelial neoplasia (crudely referred to as “cancer”). Illustrative examples are the malignant transformation of (colorectal) adenomas to carcinomas [29], loss of basal cells in prostate cancer [30], loss of myoepithelial cells in breast cancer [31], or crossing anatomical barriers, e.g., the basal layer in skin squamous carcinoma (**Fig 2C**). For those interested in modelling diagnostic AI support, knowledge of these anatomical structures is critical. The features that are truly unique to invasive cancers should be exploited—for example, the presence of features such as necrosis (dead cells) or an abundance of mitoses (dividing cells) do not imply malignancy, as they can be frequent in benign neoplasms.

**Fig 3:**
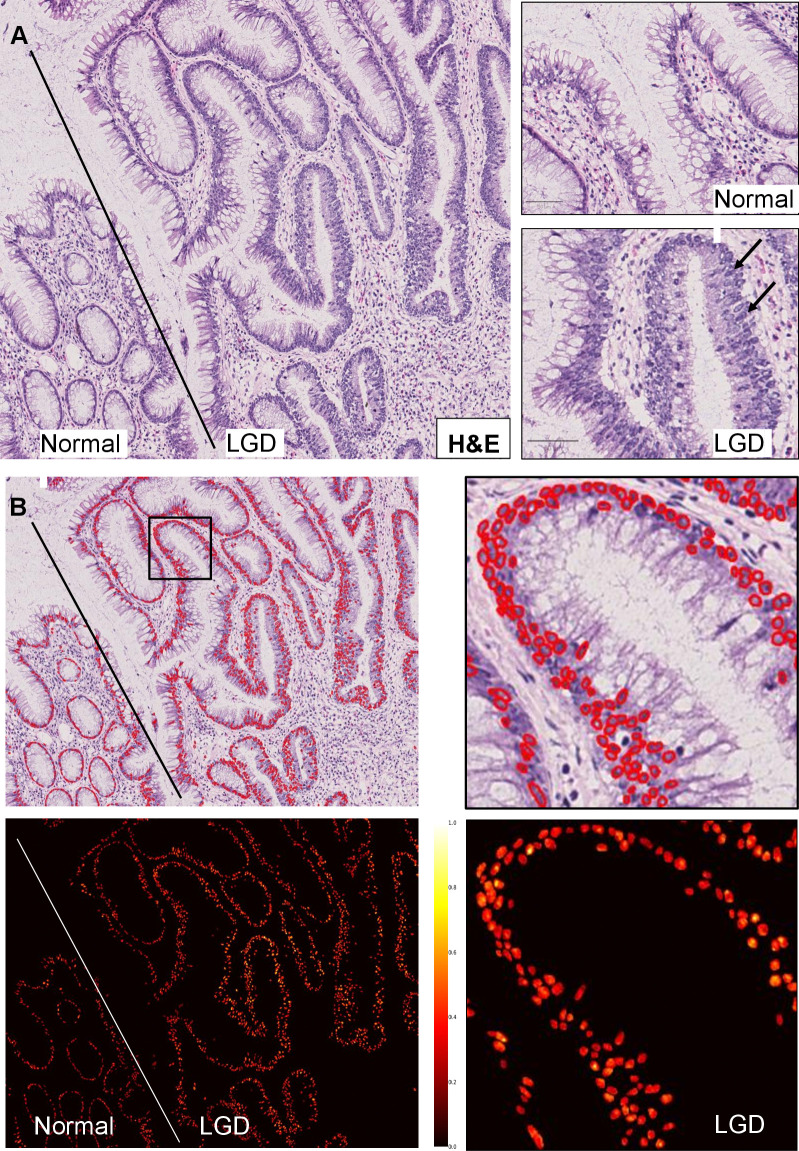
Example of computational modelling of an oncologic precursor lesion (low-grade dysplasia (LGD), human colon) with feature extraction. (**A**) Conventional features of (sporadic) colonic LGD, i.e., hyperchromasia, next to normal colonic mucosa (separated by black line) with inconspicuous enterocytes and crypts (representative example of an inflammatory bowel disease cohort, HE stain). *Arrows*: Hyperchromatic, pseudostratified (pencillate) nuclei in LGD. (**B**) Nuclei detection, produced using a deep neural network [77]. *Inset*: High magnification. (**C**) Nuclei intensity mapping. Computed from the segmentation mask in **B** by normalising the nuclei intensities across the whole slide image. LGD shows higher intensities, histologically corresponding to hyperchromasia (darker staining in HE).

Invasion is usually the final step in a sequence of malignant transformation. In epithelial tumours (e.g., gastric adenocarcinoma [32]), it is frequently preceded by, first metaplasia, and second dysplasia (definitions in **S1 Table**). Epithelial dysplasia, i.e., simply put the presence of abnormal but yet not cancerous cells [33], is a frequent precursor lesion, e.g., in upper and lower gastrointestinal tract, genital tract, skin, and the head and neck region. However, the defining morphological criteria of dysplasia differ by tissue type, and modelling approaches need to take this into account (**Table 2** and **Fig 3**). For instance, while hyperchromasia (darker staining) is a feature of dysplastic nuclei in the colon, this does not hold true for squamous dysplasia, where architectural disorders and mitoses are more diagnostic. The modeller needs a solid knowledge about which morphological criteria are defining in a respective organ, as concepts are not necessarily transferable. We provide a few examples of such features in **Table 2**.

**Table 2:** Features of epithelial tumours and strategies of incorporating them into modelling systems.

Biological object	Cellular detail	Architectural detail	Stromal detail	Additional identifiers	Relevant modelling considerations
Dysplasia in the gastrointestinal tract	Nuclear hyperchromasia [79], cell form (e.g., “pencillate” in intestinal adenomas [80])	Can be polypoid or flat	Evtl. associated inflammation		Staining intensity of epithelial cells to quantify hyperchromasia. Eccentricity, circularity, and elongation to detect pencil-like nuclei.
Dysplasia in the genital and head and neck region	Mitoses, irregular cell form, loss of cell polarity [81]	Loss of stratification of squamous epithelium	Evtl. associated inflammation	Atypical keratinisation	Detection of mitotic cells [82,83]. Cell-based morphological features (such as eccentricity, convex area, contour area, extent, perimeter, solidity, and orientation) [84]. Quantification of cell polarity [85].
Tumour invasion	Invasive “phenotype” (atypical nucleus, nucleolus), mitoses	(Partial) loss of anatomic border, e.g., a basal membrane [86]	“Desmoplasia,” potential immune response		Proportion of continuous versus disrupted basal membrane/layer, “desmoplastic” stroma [87].
Adenocarcinoma	Gland-forming, mucus	Complex clustering of carcinoma glands	“Desmoplasia,” potential immune response	“Dirty necrosis” (not specific for a cancer type) [88]	Glandular shape descriptors [89]. Nuclear shape descriptors (bizarre form, nucleolus). Mucus can be used as a proxy.
Special subtypes and growth patterns of adenocarcinoma	Signet ring cells (intracellular mucus, compressed nucleus), giant cells	e.g., (micro)papillary, tubular, acinar, lepidic	“Desmoplasia,” potential immune response		Detection of signet ring cells [90]. Detection of specific and unique growth patterns (such as lepidic, papillary, acinar, cribriform, micropapillary and solid [91]).
Squamous cell carcinoma	Intercellular bridges	Keratin “pearls,” specific growth pattern (e.g., basaloid)	“Desmoplasia,” potential immune response		Detection of keratin pearls [92]. Detection of intercellular bridges [93].

### The “face” of malignancy—Morphology and pitfalls

Not every malignancy follows the conventional benign-intermediate-malignant trajectory described above, examples being de novo (i.e., not arising from a precursor tumour, such as a skin mole (“naevus”)) malignant melanoma [34] or sarcoma (relatively rare malignant tumours of the soft tissue or bone). Briefly, malignant tumours can be classified according to their cell(s) of origin, e.g., epithelial, mesenchymal (simply put, connective tissue), or lymphoid (see above). It’s reasonable to assume that for developers and the healthcare system, epidemiologically frequent malignant tumours are most relevant, these being malignant epithelial tumours, namely (adeno)carcinomas, e.g., of the prostate, breast, colon (rectum), and lung [21]. Adenocarcinomas are a morphological (and also molecularly distinct) subset of carcinomas (e.g., in contrast to squamous cell carcinomas) and have a typical morphology with gland-like (i.e., circularly arranged with a central lumen) growth with malignant nuclear features such as bizarre cell forms, mitotic figures, and a prominent nucleolus (**Fig 2A**); however, there are always exceptions and this can make modelling tricky and algorithms incompatible with routine diagnostic practice. Illustrative exceptions are deviant subtypes or atypical cancer growth patterns, such as high-grade prostatic adenocarcinoma with diffuse growth [35], or invasive-lobular breast cancer with discohesive tumour cells [36]. Not incorporating these features into algorithms is severely limiting and might jeopardise clinical conclusion from a computer-assisted diagnostic setup. Non-small cell lung cancer (NSCLC), second most frequent cancer in both sexes in the United States, has a known multitude of growth patterns [37] and also colorectal cancer can exhibit a very deviant morphology, even if rare [38]. In addition, adenocarcinoma is frequently accompanied by a strong stromal response (“desmoplasia”), which can be seen by collagen deposition and extracellular matrix (ECM) recomposition [39] (**Fig 2B** and **Table 2**). Squamous cell carcinoma, most prominently in the head and neck region (**Fig 2C**), genital tract (cervical cancer, anal cancer) and lung is characterised by keratin “pearls” (whorl-shaped accumulations of keratin, a structural protein), and intercellular bridges (specialised connections between adjacent cells). It has to be kept in mind that even very typical diagnostic features might not be apparent in poorly differentiated tumours which have lost much of their resemblance to the tissue of origin (“dedifferentiation” is the process of losing tissue specialisation, returning to a less specialised state). Mixed carcinomas, e.g., adenosquamous, adeno-neuroendocrine (e.g., mixed neuroendocrine-nonneuroendocrine neoplasms [40]), add further to the complexity. Lastly, malignancy of other lineages, e.g., non-epithelial shows a considerable amount of variation, too. In particular, malignant melanoma is known for its plethora of “morphological faces” [41].

### Composition of, and modelling the tumour microenvironment (TME)

The TME is the complex biological ecosystem surrounding a tumour. It is composed of tumour-infiltrating lymphocytes (“TILs”) [42], (cancer-associated) fibroblasts and (tumour-associated) neutrophils (CAFs, TANs) [43,44], macrophages, extracellular matrix, and supportive elements such as microvessels. All of those have gained significant attention due to their prognostic, tumour-promoting or -suppressive impact. While macrophages have traditionally been dichotomously subclassified (i.e., M1- and M2-polarised [45]), TILs can be more deeply sub-stratified.

Modelling the TME needs a comprehensive strategy due to its inherent level of complexity and set of “players.” Nevertheless, modelling using morphology is possible to a certain degree as the “players” generally have distinct cellular and architectural features (**Table 1**). In the following, we introduce routine stainings and immunohistochemistry that can facilitate the morphological characterisation of tumours and their TME.

## Routine tissue protocols

### Formalin-fixed paraffin-embedded (FFPE) and fresh frozen (FF) tissue

Formalin-fixed, paraffin-embedded (FFPE) tissue preservation is the gold standard in histopathology for maintaining tissue integrity. This technique, first introduced by German pathologist Friedrich Blum in 1896 [46], involves fixing tissue samples in formalin, which preserves their cellular structure by cross-linking proteins. The fixed tissues are then dehydrated, embedded in paraffin wax, and formed into a solid, archivable tissue block. These blocks can be sectioned into thin slices (usually between 2 and 10 μm [47]), mounted onto glass slides, and stained allowing for microscopic examination. Fresh frozen (FF) tissue on the contrary is immediately preserved by snap-freezing at −196°C in liquid nitrogen (e.g., cancer tissue within 1 h from surgery), without (formalin) fixation. As FF is tissue in its purest form, it is more accurate for genomic analysis than FFPE. However, FFPE preserves better structural integrity and is much more standardised (and affordable) for conventional staining and immunohistochemistry (see below). Despite these advantages, it is crucial to consider that the number of (viable) cells of tissue samples is highly heterogeneous and depends not only on the tissue (and biopsy site) itself but also on how it is retrieved [48]. In general, surgical resection specimens collected in conventional FFPE tissue cassettes (approximately 1 × 1 × 0.5 cm) tend to contain the most cells, while the amount of cells in tissue biopsies is much reduced and limited by several factors such as, for instance, the gauge of the biopsy needle [49], the anatomical site (e.g., soft tissue is less cellular than bone marrow), and the expertise of the operator [50,51].

### The hematoxylin and eosin (HE) stain

The hematoxylin and eosin (HE) stain is the standard staining that has been used in (diagnostic) histopathology for many years [52]. While hematoxylin stains acidic structures, e.g., the nucleus, in different degrees of blue-purple, eosin stains basophilic structures in red-pink, such as the cytoplasm and ECM (**Fig 1**). This allows for the identification of common cell types and their arrangement in space. The HE stain is cheap, widely used and well accepted in the diagnostic community [53]. A low amount of staining variability is critical for both diagnostics and AI algorithms [54].

### Immunohistochemistry (IHC) and immunofluorescence (IF)

**Fig 4:**
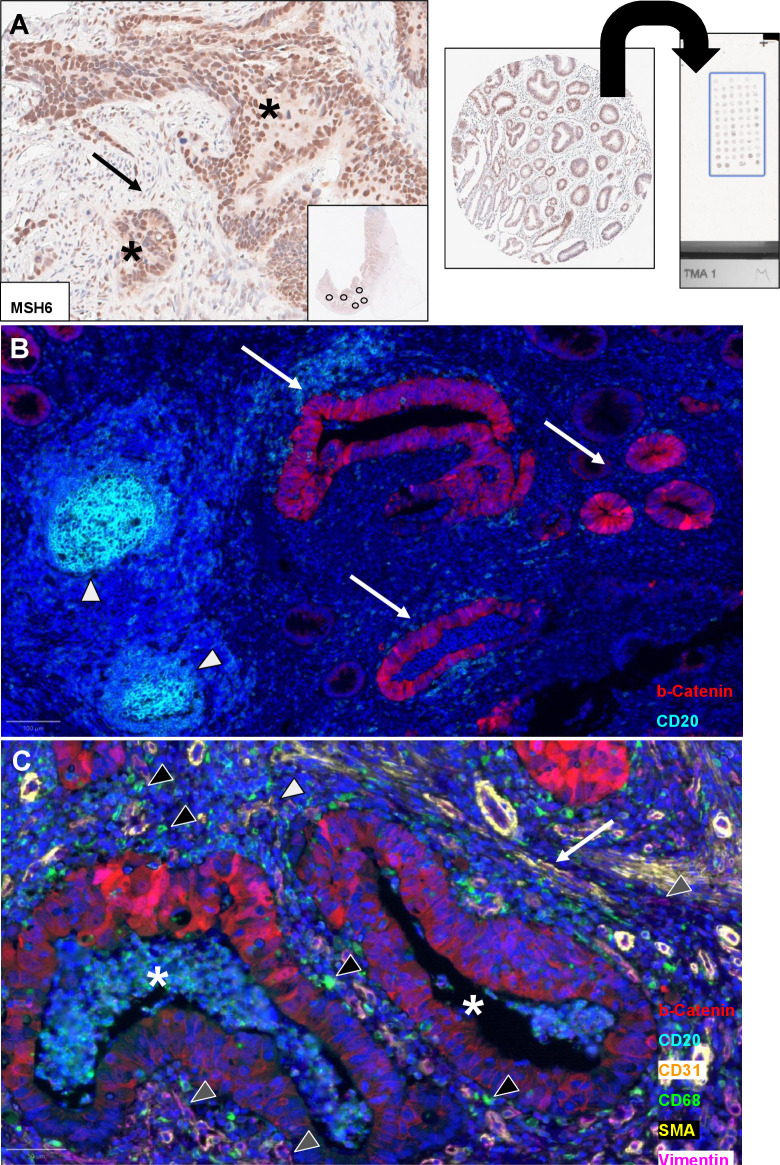
Immunohistochemistry (IHC) and multiplexing. (**A**) Singleplex IHC and tissue microarrays (TMAs). Left: Preserved nuclear expression of the mismatch repair protein MSH6 in a colorectal adenocarcinoma sample (asterisks). *Arrow*: Preserved expression in stromal fibroblasts. On the right, the principle of TMA construction: Tissue cores, usually 6 to 10 mm in diameter, are punched and transferred to a new FFPE block, which is composed of multiple patients and tissues. *Inset*: Overview of colorectal cancer tissue and illustrative punches. (**B**) Basic multiplex immunofluorescence (mIF) panel on colorectal mucosa consisting of 2 markers and DAPI (blue) as a standard nuclear stain. CD20 (light blue) labels B-cells and a lymphoid follicle (arrowheads). Beta-catenin stains colonic crypts (red, arrows). (**C**) Extended mIF panel with 6 markers and DAPI. Beta-catenin (red) highlights colorectal adenocarcinoma glands (asterisks), while the space between malignant glands is composed of immune cells, such as B-cells (CD20, light blue), and macrophages (CD68, green, black arrowheads). CD31 (orange) highlights blood vessels (grey arrowheads, intermixed with SMA, yellow) and might suggest vascular invasion. The stroma is represented by Vimentin (purple, grey arrowheads)-positive fibroblasts and smooth muscle (SMA, yellow. arrow). *CD*, Cluster of Differentiation; *SMA*, smooth muscle actin.

HE stains allow for a vast amount of tissue interpretability, but in order to address cells and their interplay more granularly, auxiliary information can be obtained from immunohistochemistry (IHC). (Single-plex) IHC has revolutionised histopathology in the 20th century [55] and continues to be an indispensable tool. In principle, IHC detects a target antigen of interest (e.g., membrane transporters, enzymes) by using a chromogen-linked commercial antibody that binds to the antigen of interest and “staining” it a particular colour—usually brown (**Fig 4A** and **Table 3**) [56]. The target of interest could be in tumour cells or in cells of the TME.

**Table 3:** Representative markers for multiplexing panels (Fig 4). *ALK*, anaplastic lymphoma kinase; *AR*, androgen receptor; *ARID1A*, AT-rich interactive domain-containing protein 1A; *Bax*, Bcl-2-associated X protein; *CA*, carboanhydrase; *CD*, Cluster of Differentiation; *CK*, cytokeratin; *EGFR*: Epidermal Growth Factor Receptor; *ER*, *oestrogen receptor*; ERG, ETS-Related Gene; *FOXP3*, forkhead box P3; *GLUT1*, glucose transporter member 1; *INSM1*, insulinoma-associated protein 1; *LCA*, leukocyte common antigen; *LDH*, lactate dehydrogenase; *MCT*, moncarboxylate transporter; *mdm2*, mouse double minute 2 homolog; *MMR*, mismatch repair; *MEK*, mitogen-activated protein kinase kinase enzyme; *MPO*, myeloperoxidase; *MUM1 (IRF4*), multiple myeloma 1 (interferon regulatory factor 4); *PD-1/PD-L1*, programmed death/ligand-1; *PHH3*, phospho-histone H3; *PgR*, progesterone receptor; *PTEN*, phosphatase and tensin homolog; *Rb1*, retinoblastoma protein; *SMA*, smooth muscle antigen; *SOX10*, SRY related HMG box 10.

Target of interest	Antigen/Marker
Basic anatomical structures	Blood/Lymphatic vessels: CD31, CD34, ERG / D2-40 (Podoplanin) Connective tissue: e.g., collagens (Smooth) muscle: Desmin, SMA Nerves: S-100, SOX10
Immune infiltrate (see **Table 1**)	B-cells: e.g., CD19, CD20 Cytokines: e.g., Interleukin/s (Il) such as Il-17 for Th17 cells Macrophages: e.g., CD11b, CD68, CD163 Neutrophils: e.g., CD15, MPO Natural Killer (NK)-cells: e.g., CD16, CD56, CD57 Plasma cells: e.g., MUM1 (IRF4) T-cells: e.g., CD3, CD4 (T-helper), CD8 (cytotoxic), CD45 (LCA), Granzyme B (activated T- and NK-cells) T-regulatory cells: e.g., FOXP3
Lineage	Epithelial: e.g., Cytokeratin/s Melanocytic: e.g., Melan-A, S-100, SOX10 Mesenchymal: e.g., Desmin, SMA Neuroendocrine: e.g., Chromogranin A, INSM1, Synaptophysin
Tumour invasion	Calponin, CK5/6, p63 (basal cell layer loss in prostate cancer, myoepithelial cell loss in breast cancer), collagens (basal membrane)
Proliferation/Apoptosis	e.g., proliferation index Ki67, PHH3 (mitoses)/Caspases (such as Caspase-3) or Bax, Fas (CD95)
Drug response and prediction	e.g., Her-2, hormone receptors (e.g., AR, ER, PgR), MMR proteins, PD1/PD-L1
Surrogates for molecular alterations	e.g., ALK, ARID1A, BRAF (V600E), EGFR, mdm2, MEK, p53, PTEN, Rb1
Hypoxia and metabolism	e.g., CA9, GLUT1, LDH, MCT1, MCT4

While IHC uses enzymes as chromogens, immunofluorescence (IF) uses fluorescent dyes (fluorophores) conjugated to antibodies. Advantages of IF are higher resolution and an improved visualisation of co-localised antigens. On the contrary, IHC stainings are long-lasting, cheaper and can be viewed by light microscopy. Some antigens are of particular relevance for diagnostic and (consequently also) AI-developing purposes, namely the proliferation marker *Ki-67* [57,58] or lineage markers such as cytokeratins [59] (**Table 3**). “Clusters of differentiation” (CDs) are surface proteins that can help with subtyping cells, particularly immune cells (refer to https://ftp.uniprot.org/pub/databases/uniprot/knowledgebase/complete/docs/cdlist.txt). This is useful as there is little potential to identify an immune cell subpopulation from an HE alone. IHC staining should be validated extensively, as some antibodies tend to cross-react among different targets leading to lack of specificity and misleading results [60]. Internal on-slide controls can be helpful as a quality control, such as cross-reacting (stromal) cells.

Tissue microarrays (TMAs) (**Fig 4A**), i.e., assembling a multitude of usually 0.6 to 1 mm sized tissue cores into a single slide, have allowed for a high-throughput setup [61]. Depending on the tissue type and anatomic site, a TMA core usually captures between a few hundred and a few thousand cells per tissue core [62]. TMAs allow multiple stainings and tissues (from different patients) can be analysed under standardised conditions [63,64].

### The advent of multiplexing

Recently, antigen visualisation reached a new era in which we can detect up to hundreds of markers of interest in one section of tissue (recent comprehensive review in [65]) (**Table 3**). Basic panels visualise cells of interest and key anatomic structures [66], for instance, an epithelial, a pan-leukocyte marker and vessels (e.g., a cytokeratin, CD45, CD31). Multiplexing allows for multiple panels which represent different compartments (**Fig 4B** and **4C** and **Table 3**) characterising the TME [67] and its neighbourhoods [68]. This increasing plexity allows for the interrogation of biology in more detail, but results in more complex data sets than HE. This could present significant challenges for AI models, which include the demand for larger training data sets (and thus higher computational power) to reach a similar level of performance seen for HE, higher likelihood of technical artefacts due to more complex wet lab protocols, and the difficulty of jointly modelling multiple cellular markers and their spatial relationships. Further, different markers often co-localise, which introduces additional difficulties to the modelling process [69]. Thus, compared to HE, analysing both IHC and IF data is usually harder and more expensive. A challenge further exacerbated by increasing plexity.

## Challenges and outlook

To be implemented into routine practice, an AI algorithm needs several indispensable properties, i.e., clinical relevance, high accuracy, rapid implementation, fast computation, and last but not least, user-friendliness. Perfect accuracy is desired for pathological diagnostics, such as differentiating between tumour invasion and benign disease; anything less could put patients’ lives at risk. False negatives that lack a prognostic biomarker may lead to reduced therapeutic options. A variety of technical difficulties, such as staining differences, scanner variability, image modalities, and image size [70,71] hinder the performance of AI models, including their generalisation to different datasets. For these models to achieve robust generalisation across different datasets, several key standardisation approaches are required throughout the imaging and analysis pipeline, such as introducing standardisation of tissue processing, sectioning thickness, reagents, fixation protocols, scanner calibration, and performing stain normalisation [72–74]. Further, multi-centre validation data sets that are able to represent real-world technical variations could also help in developing and validating more generalised AI models. Aside from the need of standardisation, the AI developer is frequently confronted with the profound problem of lacking in-depth biological, morphological, and structural knowledge. It is our hope that this work enables the developer to leverage biologically relevant features into designing computational models. It is our hope that this work enables the developer to leverage biologically relevant features into designing computational models. Further, this becomes helpful in explaining the output from “black-box” AI models, by correlating the results with known biological features. With a common knowledge level, the design of “pathologist in-the-loop” approaches [75,76] in training AI models are facilitated.

## Supporting information

S1 Table A glossary of different “-plasias” with examples. (DOCX)
