# Supplementary material for: The tumour histopathology “glossary” for AI developers
Source: PLoS Comput Biol. 2025 Jan 23;21(1):e1012708. doi: 10.1371/journal.pcbi.1012708 (PMC11756763; doi:10.1371/journal.pcbi.1012708)
Supplement: S1 Table — (DOCX) [file pcbi.1012708.s001.docx]

**Table S1.**

| **“-plasia”** | **Definition** | **Examples** |
| --- | --- | --- |
| *Hyperplasia* | Increase in the number of cells (versus “hypertrophy”, i.e. increase in cell volume) | Stromal hyperplasia (e.g. ovary), benign prostatic hyperplasia, sebaceous hyperplasia |
| *Metaplasia* | Replacement of a cell type to another cell type, usually due to a stimulus (such as, for instance, chronic irritation/inflammation) | Intestinal metaplasia (e.g. gastric [(94)](https://www.zotero.org/google-docs/?uq4Kbb), apocrine metaplasia (e.g. breast [(95)](https://www.zotero.org/google-docs/?YqwFoh)), squamous metaplasia (genital), epidermoid metaplasia |
| *Dysplasia* | “Abnormal” (= deviant from their tissue of origin in terms of size, shape, and/or organisation) cells but yet without invasive capacity | Gastrointestinal and oral dysplasia, squamous intraepithelial lesion (SIL) of the genital tract |
| *Anaplasia* | Poor cellular differentiation with no resemblance to the initial lineage | Anaplastic tumours, e.g. anaplastic astrocytoma, or as poor prognostic factor [(96,97)](https://www.zotero.org/google-docs/?AbOaKB) |
